# Supplementary material for: Resolving the full spectrum of human genome variation using Linked-Reads
Source: Genome Res. 2019 Apr;29(4):635–45. doi: 10.1101/gr.234443.118 (PMC6442396; doi:10.1101/gr.234443.118)
Supplement: Supplemental Material [file supp_gr.234443.118_Supplemental_longranger-2.2.zip › longranger-2.2.tar.bz2!/lr2.2-public/lib/stan/cmdstan/stan/doc/api/html/functions_vars_b.html]

Stan: Class Members - Variables


|  |  |
| --- | --- |
|  | Stan  2.5.0  probability, sampling & optimization |


- Main Page
- Related Pages
- Namespaces
- Classes
- Files

- Class List
- Class Index
- Class Hierarchy
- Class Members

- All
- Functions
- Variables
- Typedefs
- Enumerator
- Related Functions

- \_
- a
- b
- c
- d
- e
- f
- g
- h
- i
- k
- l
- m
- n
- o
- p
- q
- r
- s
- t
- u
- v
- w
- x
- y
- z


All Classes Namespaces Files Functions Variables Typedefs Enumerations Enumerator Friends Macros Pages

### - b -

- bare\_type\_g
  : stan::gm::functions\_grammar< Iterator >
- bare\_type\_r
  : stan::gm::bare\_type\_grammar< Iterator >
- base\_type\_
  : stan::gm::base\_var\_decl
  , stan::gm::expr\_type
- bd\_
  : stan::agrad::op\_ddv\_vari
  , stan::agrad::op\_vd\_vari
  , stan::agrad::op\_vdd\_vari
  , stan::agrad::op\_vdv\_vari
- bodies\_
  : stan::gm::conditional\_statement
- body\_
  : stan::gm::function\_decl\_def
  , stan::gm::while\_statement
- bvi\_
  : stan::agrad::op\_dv\_vari
  , stan::agrad::op\_dvd\_vari
  , stan::agrad::op\_dvv\_vari
  , stan::agrad::op\_vv\_vari
  , stan::agrad::op\_vvd\_vari
  , stan::agrad::op\_vvv\_vari


---

[ Stan Home Page ]

*© 2011–2014,
Stan Development Team.*
